# Regulation of MYB Transcription Factors of Anthocyanin Synthesis in Lily Flowers

**DOI:** 10.3389/fpls.2021.761668

**Published:** 2021-12-01

**Authors:** Xiaojuan Yin, Yibing Zhang, Li Zhang, Baohua Wang, Yidi Zhao, Muhammad Irfan, Lijing Chen, Yulong Feng

**Affiliations:** ^1^Plant Protection College, Shenyang Agricultural University, Shenyang, China; ^2^Key Laboratory of Agriculture Biotechnology, College of Biosciences and Biotechnology, Shenyang Agricultural University, Shenyang, China; ^3^Key Laboratory of Protected Horticulture (Ministry of Education), College of Horticulture, Shenyang Agricultural University, Shenyang, China; ^4^Department of Biotechnology, Faculty of Science, University of Sargodha, Sargodha, Pakistan

**Keywords:** lily, anthocyanin biosynthesis pathway, MYB transcription factor, activator, transcriptional inhibitor, promoter, structural genes

## Abstract

Flower color is the decisive factor that affects the commercial value of ornamental flowers. Therefore, it is important to study the regulation of flower color formation in lily to discover the positive and negative factors that regulate this important trait. In this study, MYB transcription factors (TFs) were characterized to understand the regulatory mechanism of anthocyanin biosynthesis in lily. Two R2R3-MYB TFs, LvMYB5, and LvMYB1, were found to regulate anthocyanin biosynthesis in lily flowers. LvMYB5, which has an activation motif, belongs to the SG6 MYB protein subgroup of *Arabidopsis thaliana*. Transient expression of *LvMYB5* indicated that LvMYB5 can promote coloration in *Nicotiana benthamiana* leaves, and that expression of *LvMYB5* increases the expression levels of *NbCHS*, *NbDFR*, and *NbANS*. VIGS experiments in lily petals showed that the accumulation of anthocyanins was reduced when *LvMYB5* was silenced. Luciferase assays showed that LvMYB5 can promote anthocyanin synthesis by activating the *ANS* gene promoter. Therefore, LvMYB5 plays an important role in flower coloration in lily. In addition, the transient expression experiment provided preliminary evidence that LvMYB1 (an R2R3-MYB TF) inhibits anthocyanin synthesis in lily flowers. The discovery of activating and inhibitory factors related to anthocyanin biosynthesis in lily provides a theoretical basis for improving flower color through genetic engineering. The results of our study provide a new direction for the further study of the mechanisms of flower color formation in lilies.

## Introduction

Lilies are bulbous perennials with large flowers that are fragrant, brightly colored and highly desired by consumers. Lily species and hybrids (genus *Lilium* in the botanical family Liliaceae) are widely used as cut flowers, and are also planted in parks, courtyards, and home gardens. It has always been the goal of lily breeding to produce lilies with colorful flowers (Liu et al., 2014). Moreover, petals are the important part of many ornamental plants, and the different colors, tones, and intensities of the petals are always due to the presence and contents of various anthocyanin pigments, which directly affects the ornamental value of plants (Alappat and Alappat, 2020). Anthocyanins, secondary metabolites produced by the flavonoid biosynthesis pathway, are found in most flowering plants (Rudall, 2020), and not only provide rich colors to flowers and other plant parts, but also prevent damage from low temperature stress, ultraviolet light, pests, diseases, and other injuries (Lotkowska et al., 2015; Liu et al., 2018).

Plant anthocyanin biosynthesis is regulated by a variety of genes (Mekapogu et al., 2020). Many structural and regulatory genes involved in anthocyanin production and accumulation have been isolated and identified (Quattrocchio et al., 1993; Xu et al., 2015). The coordinated expression of genes that encode enzymes in the flavonoid biosynthesis pathway, also called structural genes, including chalcone synthase (*CHS*), chalcone isomerase (*CHI*), flavanone 3-hydroxylase (*F3H*), flavonoid 3′-hydroxylase (*F3′H*), dihydroflavonol-4-reductase (*DFR*), and anthocyanidin synthase (*ANS*), as well as UDP-glucose:flavonoid-3-O-glucosyltransferase (*3GT*), can directly affect the biosynthesis of anthocyanins, and thus affect flower color (Nishihara and Nakatsuka, 2011; Yin et al., 2021). It has been shown that flower color formation is closely related to expression of the late structural genes *DFR, ANS*, and *3GT*, which may directly affect flower color formation in lily (Yin et al., 2020). Among these genes, *ANS*, which encodes anthocyanidin synthase, is an important enzyme that converts colorless leucoanthocyanidins into colored anthocyanidins (Jaakola, 2013; Li et al., 2019). Moreover, it was found that the structural genes can be regulated by single MYB transcription factors (TFs) or the MBW complex, which is composed of MYB, bHLH, and WD TFs (Xu et al., 2015; Lloyd et al., 2017). MYB TFs can bind to cis-acting elements of gene promotors to regulate the intensity and pattern of structural gene expression, and affects pigment accumulation both temporally and spatially (Gong et al., 2004). Therefore, MYB TFs play a decisive role in regulating anthocyanin accumulation (Naing and Kim, 2018).

In plants, the level of anthocyanin accumulation is determined by the expression levels of MYB positive/negative regulators (Song et al., 2020; Wei et al., 2020). MYB gene family subgroup 6 (SG6) members in *Arabidopsis thaliana* can regulate the expression of late anthocyanin synthesis pathway genes (Stracke et al., 2001). In *Arabidopsis thaliana* leaves and seedlings, the overexpression of *AtMYB75/90/114* leads to pigmentation (Gonzalez et al., 2008). However, the overexpression of *AtMYB3/4/7/32*, members of *A. thaliana* MYB gene family subgroup 4 (SG4), inhibit the synthesis of anthocyanins (Chen et al., 2019), showing that they are negative regulators of pigmentation (Kranz et al., 1998). Similarly, *MdMYB1/10/11*, and *MdMYBA* positively regulate pigmentation in *Malus* sp. (Ban et al., 2007; Espley et al., 2007; Mao et al., 2021), while *MdMYB16/308/111* have a negative effect on pigmentation (An et al., 2015; Liu et al., 2019a; Zhang et al., 2019). Therefore, anthocyanin synthesis is co-regulated by positive and negative transcription factors, so the identification of MYB TFs that play positive and negative regulatory roles in anthocyanin biosynthesis is important in flower color research.

MYB TFs associated with anthocyanin synthesis usually contain a conserved R2R3 domain. The R2 domain contains a conserved DNA binding site, while R3 contains the conserved domain [D/E]Lx2[K/R]x3Lx6Lx3R that can bind to bHLH proteins. Most of the MYB TFs associated with activation of anthocyanin biosynthesis contain a conserved activation domain, [K/R]P[Q/R]P[Q/R] (Munoz-Gomez et al., 2021), examples are MaAN2, AcMYB123, FaMYB10, and MdMYB10 (Espley et al., 2007; Chen et al., 2017; Wang et al., 2019; Zhang et al., 2020). All of these MYB proteins contain activation domains. Overexpression of the genes that encode these proteins can promote anthocyanin accumulation by acting on structural gene promoters (Feng et al., 2020).

In plants, MYB TFs can inhibit anthocyanin synthesis in a variety of ways (Chen et al., 2019). First, the MYB inhibitor can reduce the activity of the MBW complex to affect anthocyanin synthesis by competitively binding bHLH (Zhou et al., 2019). For example, IbMYB44 in sweet potato is an inhibitor that can interact with the MYB340-bHLH2-NaC56 complex and inhibit its activity through competitive binding of bHLH2, which negatively affects anthocyanin synthesis (Wei et al., 2020). In addition, there are some other negative MYBs that inhibit anthocyanin biosynthesis without the need for competitive binding of bHLH partners. The C1 (LIsrGIDPxT/SHRxI/L), EAR (LxLxL or DLNxxP), or TLLLFR inhibitory motifs are conserved at the C-terminal ends of these inhibitor proteins (Kranz et al., 1998; Dubos et al., 2010), and are the most common transcriptional inhibitory motifs found in plants (Kagale and Rozwadowski, 2011; Ma and Constabel, 2019). Studies have shown that the ERF-associated-amphiphilic repression (EAR) motif recruits histone deacetylases, which deacetylate the histones on chromosomes, compacting the nucleosomes, which prevents them from binding to other TFs, exerting an inhibitory effect (Pazin and Kadonaga, 1997; Thiel et al., 2004). Also, TOPLESS (TPL) proteins generally mediate transcriptional repression in numerous developmental pathways. Some MYB TFs are able to recruit EAR motif-dependent TPL co-repressors to transform activated MBW protein complexes into inhibitory complexes (Zheng et al., 2019). For example, PhMYB27 and MdMYB16 exert negative MYB activities through their EAR motifs (Albert et al., 2014; Xu et al., 2017).

Research on the TFs that regulate anthocyanin production is the theoretical basis for coloration in molecular breeding. In previous studies, LhMYB12, LhMYB12-lat, LrMYB15, and LhMYB18 were shown to regulate anthocyanin pigmentation in whole petals, ovaries, buds, and the large anthocyanin spots on lily flowers, respectively. Therefore, multiple MYB TFs can co-regulate the formation of lily flower color (Xu et al., 2016; Yamagishi, 2016, 2018). However, the positive and negative TFs that co-regulate flower color in lily have not been thoroughly studied. In particular, the negative regulators of anthocyanin biosynthesis are less studied compared to TFs that positively regulate floral pigmentation. Yin et al. (2020) carried out a detailed analysis of lily petal RNA-seq (PRJNA649743) data and characterized genes related to the regulation of lily petal coloring, including structural genes and MYB transcription factor genes. The FPKM value of *LvMYB5* (TRINITY_DN103447_c0_g1) was increased during lily flower coloration, and was possibly promoting the coloring of lily flowers. In addition, we found that the FPKM values for *LvMYB1* (TRINITY_DN96669_c0_g6) showed a declining trend during lily flower coloration, which may have a negative effect on petal coloration. However, the functions of these genes have not been verified in previous studies. In this study, the genes encoding two MYB TFs, *LvMYB1*, and *LvMYB5*, that are related to lily flower color formation, were cloned, and their functions were characterized. The functions of the proteins encoded by these genes were verified by transient overexpression, virus-induced gene silencing (VIGS), a double luciferase assay, and subcellular localization. Thus, in this study, we analyzed the regulatory effects of two different MYB genes in lily flower pigmentation to reveal the molecular regulatory mechanisms that play roles in lily flower color formation. This will give us a better understanding of the development and regulation of flower color in lily and other ornamental plants.

## Materials and Methods

### Plant Materials

In this study we used an oriental hybrid lily (cultivar “Vivian”). Plants were grown in a greenhouse at 2–25°C at Shenyang Agricultural University in 2020. Petals in the bud period (S1), coloring period (S2), and full-bloom period (S3) were collected and flash frozen in liquid nitrogen. The samples were then stored at –80°C. *Nicotiana benthamiana* seedlings were used in experiments at 4–6 weeks of age.

### Measurement of Petal Color

Total anthocyanins were extracted and the concentrations were determined using the method described in Shin et al. (2007). Briefly, 0.1 g tissue samples were ground to a powder in a mortar with liquid nitrogen and incubated in 600 μl of extraction buffer (methanol containing 1% HCl) overnight at 4°C in the dark. After extraction, 400 μl of water and 400 μl of chloroform were added to each sample to remove the chlorophyll. The samples were then centrifugated at 14,000xg for 5 min at 4°C to sediment the plant material, and the absorbance of the aqueous solution was read at 530 and 657 nm with a spectrophotmeter. The anthocyanin content was determined using the following equation: A_530_–0.33 × A_657_, and each sample was extracted and measured in three independent experiments. SPSS16.0 software was used for data analysis.

### Isolation and Cloning of *LvMYB1* and *LvMYB5* and MYB Protein Sequence Alignment

RNA was extracted from petals of lily flower. The improved CTAB (cetyl trimethylammonium bromide) method was used for RNA extraction. First-stand cDNA was synthesized using reverse transcriptase (Vazyme, Nanjing, China). *LvMYB1* and *LvMYB5* sequences were obtained from the database (NCBI project: PRJNA649743). *LvMYB5* and *LvMYB1* were cloned from cDNA of the lily cultivar “Vivian” petals. PCR amplification was conducted using Taq DNA Polymerase and the primers listed in Supplementary Table 1. The amino acid sequences were translated using DNAMAN software. A Maximum Likelihood tree was constructed using MEGA version 7.0 to analyze the phylogenetic relationships among MYB proteins (Kumar et al., 2016).

### qRT-PCR Analysis

qRT-PCR assays were performed using SYBR Green Master Mix (Vazyme, Nanjing, China) on a Bio-Rad CFX96^TM^ Real-Time system (Bio-Rad Laboratories, Inc., Hercules, CA, United States) in triplicate. The 10 μl reaction mixes contained 0.5 μl of each gene-specific primer and 2 μl of first-strand cDNA diluted 10-fold. The amplification program was as follows: 95°C for 5 min, followed by 40 cycles of 95°C (10 s) and 60°C (25 s). *GAPDH* and *Actin* were used as the reference genes. Relative gene expression was calculated using the 2^–ΔΔCt^ method, where ΔCt = Ct (target gene)-Ct (actin). The relative fold-change values of three biological replicates were used to calculate the mean values and standard errors (Pfaffl, 2001). The primers used in the experiment are given in Supplementary Table 1.

### Transient Overexpression of MYB Genes in *Nicotiana benthamiana* Leaves and Lily Petals

The LvMYB5 and LvMYB1 cDNAs were cloned into the pGreenII 0029 62-SK vector between the *Bam*HI and *Eco*RI sites under the control of the 35S promoter. The plasmid constructs were transformed into *A. tumefaciens* strain GV3101 along with the pSOUP helper plasmid that is required for replication of the pGreenII vector using the freeze-thaw method, and the plates were incubated at 28°C for 2 days. The resulting constructs were named 35S:*LvMYB5* and 35S:*LvMYB1*, and the sequences of the primers used are given in Supplementary Table 1.

Transient overexpression assays were carried out in leaves of the wild tobacco relative *Nicotiana benthamiana* (Voinnet et al., 2003; Hellens et al., 2005). Briefly, a single Agrobacterium colony was inoculated into 10 mL of liquid LB medium supplemented with the appropriate antibiotics and shaken until the A_600_ reached 0.8–1.0. After centrifugation, the cell pellets were resuspended in buffer (10 mM MES, 10 mM MgCl_2_, 150 mM acetosyringone, pH 5.6) at an A_600_ of exactly 0.7 and were infiltrated into the leaves of 6-week-old *Nicotiana benthamiana* plants and the outer petals of lily flower buds (about 10 cm), which were green and just beginning to color at this stage. The Agrobacterium culture carrying the empty pGreenII 0029 62-SK vector was the negative control. Transient expression assays were performed with three biological replicates, and there were six plants per replicate. The infiltrated plants were placed in a growth chamber at 24°C and 60% humidity with a 16 h light/8 h dark photoperiod for phenotype identification. Digital photographs of the leaf and petal color phenotype were taken 1 week after infiltration.

### Virus-Induced Gene Silencing

A 330 bp fragment of *LvMYB5* was PCR amplified from a specific region (Supplementary Figure 1) with the gene-specific primer pair *LvMYB5*-VIGSF and *LvMYB5*-VIGSR, which had *Eco*RI and *Xho*I sites appended to the 5′ termini of the forward and reverse primers, respectively (Supplementary Table 1). This fragment was subsequently cloned into the pTRV2 vector. The recombinant plasmid pTRV2-*LvMYB5* was transformed into *E. coli* DH5α, and the colonies were PCR-screened to select positive clones for DNA sequencing. The verified recombinant plasmids were transformed into *Agrobacterium tumefaciens* strain GV3101 using the freeze-thaw method (Yan et al., 2012).

Agrobacterium colonies carrying the pTRV-VIGS vectors (pTRV1, pTRV2, and pTRV2-*LvMYB5*) were shaken at 28°C in LB medium containing 50 mg/L rifampicin and 50 mg/L kanamycin until the A_600_ reached 0.8–1.0. After centrifugation, the cells were resuspended in buffer (10 mM MES, 10 mM MgCl_2_, 150 mM acetosyringone, pH 5.6) at an A_600_ of exactly 0.7 and incubated at room temperature for 3 h. Before infiltration, equal volumes of agrobacterial cultures carrying pTRV1 and pTRV2 (or pTRV2 derivates) were mixed. The infiltration mixtures were introduced into the outer petals of the coloring bud with a 1-mL needleless syringe until the water-soaked area accounted for ∼50% of the petal surface. Each treatment consisted of six biological replicates. The infiltrated plants were placed in a growth chamber (24°C, 60% humidity, 16 h light/8 h dark) for phenotypic identification. Digital photographs were taken 1 week after agroinfiltration.

### Dual Luciferase Transient Assay

The *ANS* gene promoter (ANSp) was isolated from DNA libraries of lily petal using gene specific primers (Supplementary Table 1), and the cis-acting elements were predicted using the PlantCARE^1^ and PLACE (Plant Cis-acting Regulatory DNA Elements)^2^ databases. We made three deletion fragments of ANSp (1,500 bp): ANSp1 (1,200 bp), ANSp2 (900 bp), and ANSp3 (300 bp) were individually cloned into the *Sal*I and *Hin*dIII sites in the pGreenII 0800-Luc vector. The LvMYB5 (or LvMYB1) TF constructs were mixed with the promoter sequence constructs in a 1:9 (v/v) ratio and then injected into young *N. benthamiana* leaves for transient co-transformation expression analysis (Hellens et al., 2005). The ratio of transactivation activities of firefly luciferase and *Renilla* luciferase was tested using the Dual-Luciferase Reporter Assay System (E1910, Promega, United States) following the manufacturer’s instructions.

### Subcellular Localization Analysis

To create the *LvMYB5*-GFP and *LvMYB1*-GFP fusion construct, the full-length cDNAs of *LvMYB1* and *LvMYB5* (without the termination codon) were amplified and subcloned into the pCAMBIA1301-GFP vector between the *Kpn*I and *Xba*I restriction sites. The control vector and the recombinant vectors were introduced into *N. benthamiana* leaves by agroinfiltration following the method described above. The sequences of the primers used are given in Supplementary Table 1. At 72 h after infiltration, the GFP fluorescence signals in leaves were observed under a confocal laser scanning microscope (Liu et al., 2019b).

### Statistical Analysis

SPSS16.0 software (SPSS Inc., Chicago, IL, United States) was used for data analysis. All the data presented in this study were the mean values of three replicates, and are shown as mean ± SD (*^∗^p*-value ≤ 0.1, *^∗∗^p*-value ≤ 0.05, *^∗∗∗^p*-value ≤ 0.01). Different letters (a, b, c) indicate statistically significant differences between the samples.

## Results

### Anthocyanin Accumulation Is Related to Gene Expression in Lily Petals

**FIGURE 1:**
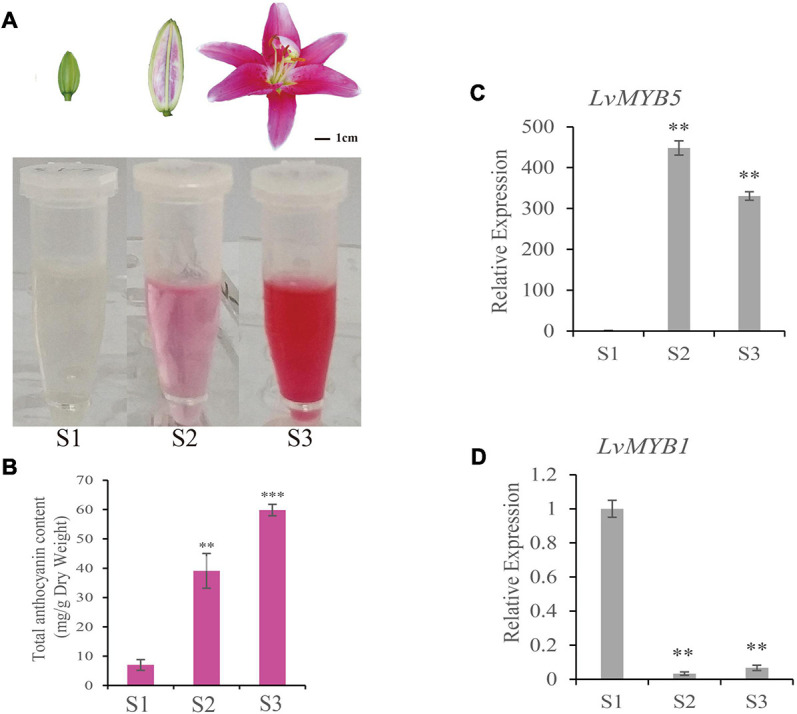
Determination of anthocyanin content and *LvMYB5* and *LvMYB1* expression analysis in lily petals during lily flower development. **(A)** Extraction of anthocyanins from lily petals at three different developmental stages (S1, S2, and S3). **(B)** Quantification of anthocyanin contents in lily petals at developmental stages S1, S2, and S3. **(C,D)** Relative gene expression levels of *LvMYB5* **(C)** and *LvMYB1* **(D)** at stages S1, S2, and S3. Data are means (± SD) of three biological replicates per variety (*^∗∗^p*≤ 0.05, *^∗∗∗^p* ≤ 0.01).

To measure the color changes during lily petal development, we measured the anthocyanin content in petals from the bud period (S1), coloring period (S2), and full-bloom period (S3). The petals from the S1 stage were colorless. S2-stage petals were partially colored, and the S3 flowers were fully opened and the petals were completely colored. Total anthocyanins were extracted from the petals at each of the three stages. The results showed that the anthocyanin content of S1 petals was extremely low compared to the other two samples (Figures 1A,B). The anthocyanin accumulation was obvious in S2 stage petals, and the anthocyanin content in S3 stage petals was the highest (Figures 1A,B). Therefore, the anthocyanins and their components gradually accumulate during flower development in lily.

In order to analyze the genes related to lily flower coloration, two regulatory genes, *LvMYB1* and *LvMYB5*, were isolated and cloned. The sequences were deposited in GenBank under accession numbers  (*LvMYB1*) and MZ882486 (*LvMYB5*). qRT-PCR assays were used to quantify gene expression during flower development. We found that, in stages S2 and S3, the expression of *LvMYB5* was up-regulated (Figure 1C), which was similar to the trend of anthocyanin accumulation (Figure 1B). The expression of *LvMYB1* was highest at the S1 stage, and was significantly down-regulated during stages S2 and S3 (Figure 1D). Therefore, *LvMYB5* and *LvMYB1* show contrasting expression patterns during petal color development. These results indicate that the two MYB TFs are both expressed during anthocyanin accumulation, but their expression patterns are very different. Therefore, they may have opposite effects on anthocyanin synthesis and/or accumulation.

### Functional Analysis of the Transcription Factors *LvMYB5 and LvMYB1*

**FIGURE 2:**
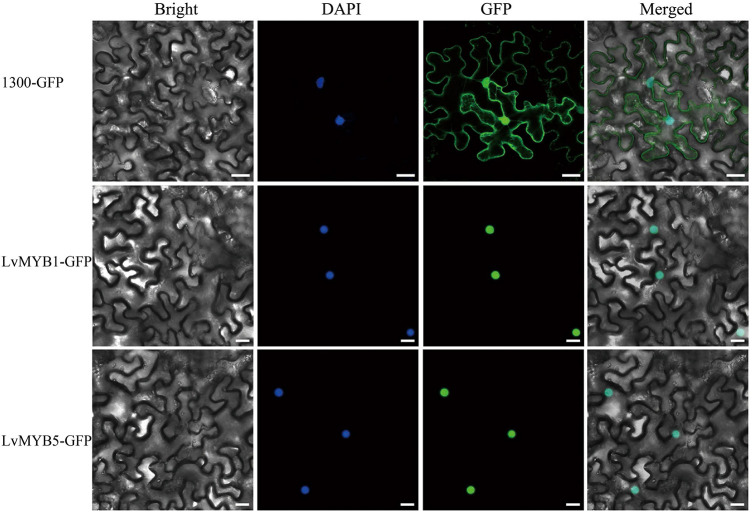
Subcellular localization of LvMYB5-GFP and LvMYB1-GFP in leaf epidermal cells of *Nicotiana benthamiana*. (Scale bars = 25 μm). Nuclei were counterstained with DAPI (4′,6-diamidino-2-phenylindole). Experiments were repeated three times.

In order to explore gene expression during petal pigmentation in lily, we cloned the *LvMYB5* and *LvMYB1* genes. The full length cDNAs are 720 and 630 bp in length, respectively. Both cDNAs contained a complete open reading frame. Subcellular localization experiments showed that the LvMYB1 and LvMYB5 proteins localize to the nucleus and are nuclear proteins (Figure 2).

**FIGURE 3:**
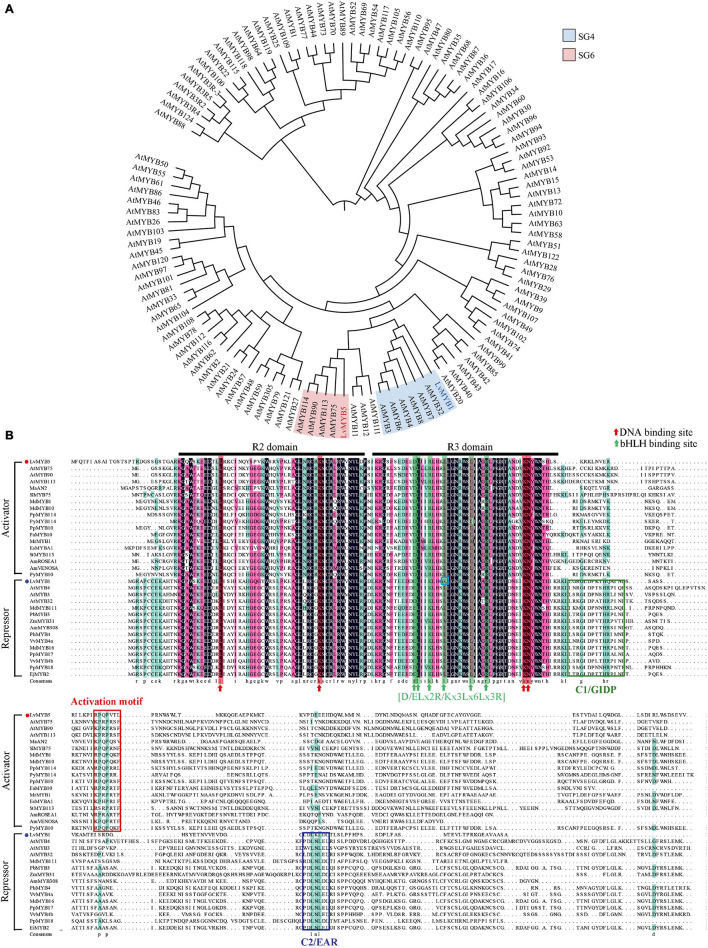
Phylogenetic tree analysis and multi-sequence alignments of LvMYB1, LvMYB5, and other MYB TF proteins related to anthocyanin biosynthesis. **(A)** A phylogenetic tree showing the evolutionary relationships between LvMYB5, LvMYB1, and MYB family proteins from *Arabidopsis*. **(B)** Multi-sequence alignments of LvMYB5 and LvMYB1 with MYB activators and repressors related to anthocyanin synthesis from other plants species. Black lines: R2 and R3 domains. Red arrow: DNA binding sites. Green arrow: bHLH binding motif. Blue box: The mutation site. Red box: The activation motif. The other colored boxes: C1/GIDP and C2/EAR motifs.

We next performed a phylogenetic analysis of the predicted protein sequences from *LvMYB1, LvMYB5* and the MYB gene family proteins from *Arabidopsis thaliana* (Figure 3A). The phylogenetic tree showed that LvMYB5 is a member of the clade containing AtMYB75, AtMYB90, AtMYB113, and AtMYB114 from the *Arabidopsis* MYB TF SG6 subgroup. Therefore, LvMYB5 may share similar functions with these proteins, which are activators of plant coloration. In addition, LvMYB1 grouped with AtMYB3, AtMYB7, and AtMYB32 in the SG4 subgroup of *Arabidopsis* MYB family TFs, and LvMYB1 may have a similar function in inhibiting anthocyanin synthesis. Therefore, we preliminarily confirmed that LvMYB5 and LvMYB1 are closely related evolutionarily to other MYB TFs that function in the regulation of anthocyanin biosynthesis.

The protein sequences of LvMYB5 and LvMYB1 were then used to construct a multiple sequence alignment with anthocyanin synthesis-related MYB TFs from other plants (Figure 3B). It was found that LvMYB5 is similar to activator-type MYB TFs from many plants, such as MaAN2, SIMYB75, AtMYB75, and MdMYB1, which contain common conserved R2/R3 domains and an activation motif. In addition, the six essential amino acids in the [D/E]Lx2[K/R]x3Lx6Lx3R motif in the R3 domain, which interact with bHLH proteins, were also conserved. Thus, LvMYB5 may function as an activator MYB TF to promote the biosynthesis of anthocyanins in lily flowers.

The multiple sequence alignment analysis of LvMYB1 and other plant MYB TFs related to anthocyanin synthesis (Figure 3B) shows that LvMYB1 is similar to MYB TFs that function as negative regulators, such as AtMYB32 and MdMYB16, which have similar R2/R3 domains and a C-terminal EAR motif (LxLxL or DLNxxP). Therefore, LvMYB1, a new R2R3-MYB inhibitor from lily, may also share similar functions with AtMYB32 and MdMYB16 to inhibit the synthesis of anthocyanins in lily flowers. However, the fourth position L (leucine) in the amino acid motif [D/E]Lx2[K/R]x3Lx6Lx3R was changed to K (lysine), which is likely to affect the interaction between LvMYB1 and bHLH TFs in the regulatory process.

### *LvMYB5 and LvMYB1* Promote and Inhibit Anthocyanin Accumulation in *Nicotiana benthamiana* Leaves, Respectively

**FIGURE 4:**
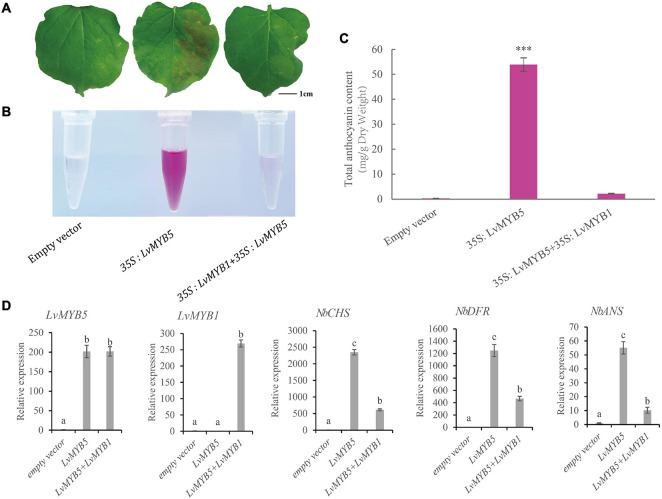
Expression analysis of *LvMYB1* and *LvMYB5* in *Nicotiana benthamiana* leaves. **(A)** Transient overexpression of *LvMYB1* and *LvMYB5* in *Nicotiana benthamiana* leaves. **(B)** Extraction of total anthocyanins from the *N. benthamiana* leaves in **(A)**. **(C)** Total anthocyanin contents in *Nicotiana benthamiana* leaves agroinfiltrated with different gene constructs. Data are means (± SD) of three biological replicates per construct (^∗∗∗^*p* ≤ 0.01). **(D)** Expression levels of structural genes and *MYB* gene in *Nicotiana benthamiana* leaves. Different letters indicate statistically significant differences between the samples.

The functions of *LvMYB5* and *LvMYB1* in anthocyanin biosynthesis were verified in a transient expression experiment. The 35S:*pGreenII* (empty vector), 35S:*LvMYB5*, and 35S:*LvMYB1* + 35S:*LvMYB5* constructs were agroinfiltrated into leaves of *N. benthamiana*. We found that the empty vector negative control did not promote coloration in the infiltrated leaves. However, there was significant accumulation of anthocyanin in the leaf epidermal cells infiltrated with the 35S:*LvMYB5* (Figure 4A). Also, there was no obvious accumulation of anthocyanin in the *N. benthamiana* leaves co-infiltrated with 35S:*LvMYB1* + 35S:*LvMYB5* (Figure 4A) and 35S:*LvMYB1*, and the relative expression of the structural genes was reduced (Supplementary Figure 2). Therefore, our results show that *LvMYB1* and *LvMYB5* have opposite effects on lily petal coloration. *LvMYB5* promotes coloration, while *LvMYB1* inhibits anthocyanin accumulation.

We extracted anthocyanins from the infiltrated *N. benthamiana* leaves and found that there was no significant pigment present in the negative control leaves. From the 35S:*LvMYB5*-infiltrated leaves, pink pigments were extracted. The *N. benthamiana* leaves infiltrated with both the 35S:*LvMYB1* and 35S*:LvMYB5* constructs contained very little anthocyanin (Figure 4B). The results of anthocyanin concentration measurements also showed that the total anthocyanin content was higher in the leaves infiltrated with 35S*:LvMYB5*, and significantly lower in the leaves infiltrated with the 35S:*LvMYB1* and 35S*:LvMYB5* constructs together (Figure 4C).

We further verified gene expression in *N. benthamiana* leaves by qRT-PCR (Figure 4D). We found that there was no significant difference in the expression of *LvMYB5* when it was overexpressed by itself or together with the *LvMYB1* construct. This indicates that *LvMYB1* does not inhibit the expression of *LvMYB5*. Analysis of the structural gene expression levels in *N. benthamiana* leaves showed that transient overexpression of *LvMYB5* caused up-regulated expression of *NbCHS, NbDFR*, and *NbANS* to different degrees compared with the negative control. When *LvMYB5* and *LvMYB1* were overexpressed simultaneously, expression of *NbCHS, NbDFR*, and *NbANS* in *N. benthamiana* was down-regulated compared with leaves that were only infiltrated with the 35S*:LvMYB5* construct. Therefore, *LvMYB5* is an activator of anthocyanin biosynthesis. On the contrary, LvMYB1 may inhibit the expression of genes in the anthocyanin biosynthesis pathway.

### *LvMYB5 and LvMYB1* Promote and Inhibit Anthocyanin Accumulation in Lily Petals, Respectively

**FIGURE 5:**
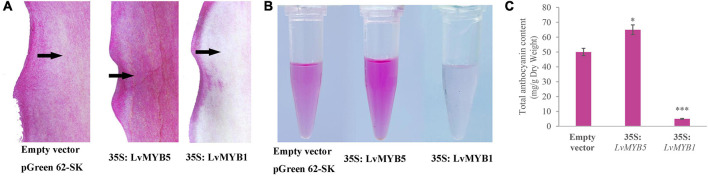
Transient overexpression of *LvMYB5* and *LvMYB1* in lily petals. **(A)** Transient overexpression changes the color of lily petals. **(B)** Extraction of total anthocyanins from lily petals after treatment. **(C)** Determination of total anthocyanin content in lily petals (*^∗^p* ≤ 0.1, *^∗∗∗^p* ≤ 0.01).

Transient overexpression experiments were carried out to investigate the functions of *LvMYB1* and *LvMYB5* on lily petal coloration. Agrobacterium cultures harboring 35S:*pGreenII* (empty vector), 35S*:LvMYB5*, and 35S*:LvMYB1* were used to infiltrate the outer petals of lily buds (about 10 cm), which were green and had just begun to color (Supplementary Figure 3). One week later, we found that the petals infiltrated with the empty vector culture were slightly discolored due to the influence of bacterial infection. However, lily petals overexpressing the 35S*:LvMYB5* construct were darker in color compared to the empty vector control petals (Figure 5A), and had higher anthocyanin levels (Figures 5B,C). In addition, the lily petals overexpressing 35S*:LvMYB1* showed obvious fading (Figure 5A), and anthocyanin accumulation was also significantly reduced (Figures 5B,C). These results further show that LvMYB5 can promote anthocyanin accumulation, while LvMYB1 can inhibit anthocyanin accumulation.

### Silencing the *LvMYB5* Gene Inhibits Anthocyanin Synthesis in Lily Petals

**FIGURE 6:**
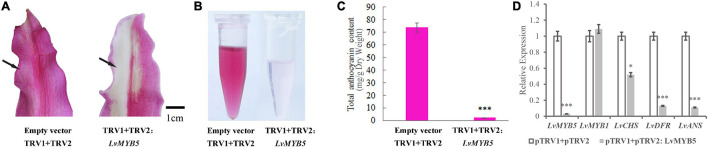
Verification of the function of *LvMYB5* by virus-induced gene silencing (VIGS). **(A)** Lily petals after treatment. **(B)** Extraction of total anthocyanins from lily petals after treatment. **(C)** Determination of total anthocyanin contents in lily petals (^∗^*p* ≤ 0.1, ^∗∗∗^*p* ≤ 0.01). **(D)** Expression levels of structural genes and *MYB* gene in lily petals.

In order to further analyze the function of the *LvMYB5* activator in anthocyanin biosynthesis in lily petals, a 330 bp DNA fragment of the *LvMYB5* gene was cloned into the pTRV2 vector to perform VIGS (virus-induced gene silencing). The cloned fragment was a specific region from the 3′ end of *LvMYB5* (Supplementary Figure 1) that avoided the conserved R2/R3 domain region of the MYB gene family. Petals from the stage when the lily buds show color were infected with the recombinant TRV construct. The results showed that the color in lily petals infected with the empty TRV vector was not significantly affected, while the color of petals infected with the pTRV2-*LvMYB5* vector became bleached after the *LvMYB5* gene was silenced (Figure 6A). Anthocyanin extraction and quantification showed that the amount of anthocyanin was significantly reduced in the region of the petals infected with pTRV2-*LvMYB5* (Figures 6B,C). Analysis of the gene expression levels in lily petals infected with the pTRV1 and the pTRV2-*LvMYB5* vector showed that it caused down-regulated expression of *LvMYB5*, as well as *LvCHS, LvDFR*, and *LvANS* to different degrees compared with the negative control, while the expression of *LvMYB1* was not significantly affected (Figure 6D). The accumulation of anthocyanin in lily petals was severely reduced when *LvMYB5* was silenced. Therefore, the silencing of *LvMYB5* can affect the pigmentation of lily petals by downregulating the expression of structural genes in the anthocyanin biosynthesis pathway. We conclude that *LvMYB5* has an important function in lily petal coloration.

### *LvMYB5* Promotes Anthocyanin Biosynthesis by Activating Anthocyanidin Synthase Gene Promoter

**FIGURE 7:**
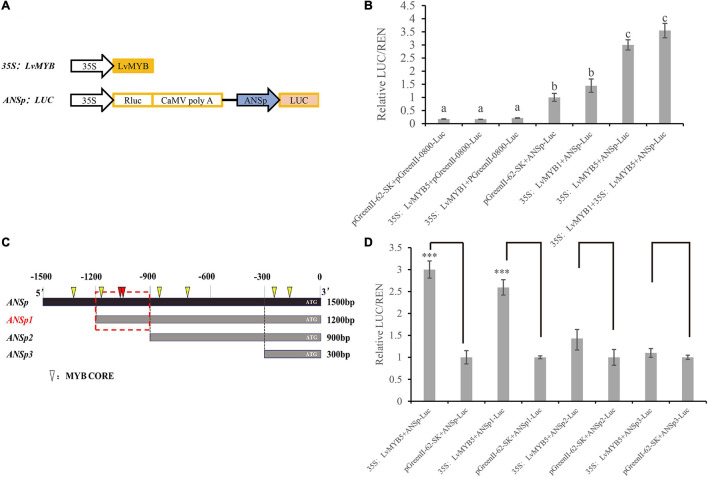
Activation of the ANS promoter by MYB TFs in lily petals. **(A)** Schematic diagrams showing the vector constructs. **(B)** Activation of ANSp by transcription factors was verified using the dual luciferase assay. Different letters indicate statistically significant differences between the samples. **(C)** ANSp fragments used to identify the active MYB TF binding sites. Triangles: MYB CORE. Red triangles: overlapping MYB CORE. Red boxes: possible binding regions. **(D)** Activation of promoter ANSp fragments by the LvMYB5 TF in lily petals. Data are means (± SD) of three biological replicates per construct (*^∗∗∗^p* ≤ 0.01).

In a previous study, we isolated a 1,500 bp DNA fragment containing the lily *ANS* gene promoter (ANSp) sequence. In order to study the function of MYB TFs, and to determine whether MYB TFs can affect anthocyanin biosynthesis by binding to the promoter of the *ANS* gene, we constructed a dual luciferase expression vector (Figure 7A). Compared to the luciferase activity from simultaneous expression of pGreenII-62-SK and ANSp-Luc in *N. benthamiana* leaves, the activity was significantly increased when 35S:*LvMYB5* and ANSp-Luc were expressed simultaneously. This indicates that LvMYB5 TF can activate ANSp. When the 35S:*LvMYB1* + ANSp-Luc constructs were infiltrated together into *N. benthamiana* leaves, the luciferase activity did not increase significantly. When 35S:*LvMYB5*, 35S:*LvMYB1*, and ANSp-Luc were co-infiltrated, the activity showed a significant increase compared to 35S:*LvMYB1* and ANSp-Luc, but there was no significant difference compared to the 35S:*LvMYB5* + ANSp-Luc treatment (Figure 7B). Therefore, the LvMYB5 TF can significantly activate the ANSp promoter to promote anthocyanin synthesis *in vivo*. In contrast, the LvMYB1 TF did not significantly affect the activity of ANSp. LvMYB1 may inhibit the accumulation of anthocyanins in other ways related to anthocyanin biosynthesis.

### Binding of the *LvMYB5* Transcription Factor to the Anthocyanidin Synthase Promoter

By analyzing the cis-acting elements in the 1,500 bp ANSp sequence, we found that there were multiple MYB binding sites in the ANS gene promoter region (Figure 7C). In order to investigate the main MYB binding sites in ANSp, we constructed expression vectors in which the ANSp DNA fragment was truncated. Based on the MYB binding site analysis, we constructed three vectors containing truncated ANS promoter fragments: the lengths of the ANSp1 ANSp2, and ANSp3 DNA fragments were 1,200, 900, and 300 bp, respectively (Figure 7C). The activation experiment conducted with the truncated ANSp fragments showed that the activity was not significantly different between the pGreenII 62-SK + ANSp2-Luc and 35S:*LvMYB5* + ANSp2-Luc treatments. Similarly, there was no significant difference between the 35S:*LvMYB5* + ANSp3-Luc and pGreenII 62-SK + ANSp3-Luc treatments (Figure 7D). Therefore, the MYB binding sites in ANSp2 and ANSp3 do not play a major role in ANS gene promoter activation by LvMYB5. However, the luciferase activities were also not significantly different between the 35S:*LvMYB5* + ANSp1-Luc and 35S:*LvMYB5* + ANSp-Luc treatments, and both of them were much higher than in any of the other treatments. These results indicate that the MYB binding sites in the –1,200 to –900 bp region of ANSp may be the main LvMYB5 binding sites; LvMYB5 can increase the activity of the promoter to induce gene expression and in turn promote the synthesis of anthocyanins in petal tissue. In addition, there are three binding sites that can be recognized by MYB TFs in this region; two of the MYB binding sites partially overlap at –1,061 bp (shown in red in Figure 7C), which may enhance their effects. Thus, it is likely that these are the main cis-acting elements that bind LvMYB5 to activate transcription of the *ANS* gene.

## Discussion

### *LvMYB5* Plays an Indispensable Role in the Pigmentation of Lily Petals

Among the various classes of transcription factors, R2R3 MYB TFs play a leading role in regulating plant pigmentation (Naing and Kim, 2018). In this study, we found that the LvMYB5 TF, which contains conserved R2 and R3 domains and an activation domain, functions as an activator of the anthocyanin biosynthesis pathway in lily flowers (Figure 3B). *LvMYB5* is similar to *MdMYB10*, *PyMYB114*, and *SlMYB75*, because the overexpression of these genes also induces anthocyanin accumulation (Espley et al., 2007; Yao et al., 2017; Jian et al., 2019). For example, there was a strong correlation between the expression of *MdMYB10* and apple anthocyanin levels during fruit development. It was suggested that the MdMYB10 TF is responsible for controlling anthocyanin biosynthesis in apple fruit. In addition, the function of *PyMYB114* was verified by transient expression in *N. benthamiana* leaves, strawberries, and pear fruits, resulting in anthocyanin synthesis. On the contrary, suppression of *PyMYB114* could inhibit anthocyanin biosynthesis in red-skinned pears. Similarly, in tomato fruits, *SlMYB75* also functions in promoting anthocyanin accumulation. All these genes contain R2 and R3 domains and an activation domain responsible for their role in anthocyanin biosynthesis.

In this study, the expression level of *LvMYB5* during stage S2 was higher than in stage S3 (Figure 1C), but the anthocyanin content in the S2 stage petals was less than in the S3 stage petals (Figures 1A,B). Anthocyanin content may be affected by gene expression, enzyme activity, and metabolic synthesis rate (Chen et al., 2012; Gao et al., 2015; Liu et al., 2019c). In the anthocyanin biosynthesis pathway, transcription factors can regulate the expression of structural genes, which encode the related enzymes (Petroni and Tonelli, 2011). TFs promote the expression of structural genes. The up-regulation of structural gene expression increased the activity of the encoded enzymes, which increased the rate of synthesis of metabolic substances. On the contrary, slightly down-regulated gene expression reduced the activity of encoded enzymes, which reduced the synthesis rate of metabolites. However, the metabolic substances were still undergoing synthesis and accumulation; only the synthesis rate was changed. In this study, the *LvMYB5* expression level increased significantly, which promoted anthocyanin synthesis in the S2 stage flower bud. With flower development, the *LvMYB5* expression level was slightly down-regulated in the S3 stage, but did not reach a significant level, which may reduce the activity of enzymes in anthocyanin biosynthesis pathway, and anthocyanin accumulated at a lower rate. This result is similar to the research on the scent compounds and related gene expression in *Clarkia breweri* flowers and *Rosa hybrida* (Dudareva and Pichersky, 2000; Chen et al., 2015; Liu et al., 2020). In addition, the regulatory network of metabolite biosynthesis is complex and diverse. A variety of TFs may also act as potential activators or inhibitors of structural genes, which may function on downstream genes by forming feedback inhibition loops to affect metabolite biosynthesis (Ye et al., 2015).

Transient expression experiments showed that *LvMYB5* can promote pigment accumulation in *N. benthamiana* leaves. In addition, *NbCHS*, *NbDFR*, and *NbANS* expression levels were up-regulated in the pigmented parts of the *N. benthamiana* leaves. Similar studies have been conducted in grape hyacinth (*Muscari armeniacum*) and showed that ectopic expression of *MaAN2* promotes pigmentation in the leaves and flowers of *N. benthamiana*. In addition, transient overexpression of *MaAN2* strongly activated the promoters of the *MaDFR* and *MaANS* genes, and promoted the accumulation of anthocyanins (Chen et al., 2017).

When the *LvMYB5* gene was silenced, gene expression in the anthocyanin biosynthesis pathway was significantly down-regulated, the synthesis of anthocyanin was down-regulated, and the color of petals infected with the pTRV2-*LvMYB5* vector became bleached. Therefore, we conclude that *LvMYB5* plays an indispensable role in the coloration of lily petals. Our results are similar to those found in other plants, such as red-fleshed kiwifruit. Suppression of *AcMYB10* expression by VIGS lead to delayed pigmentation in fruit, and the expression levels of *AcLDOX* and *AcF3GT* were significantly down-regulated. This demonstrated that *AcMYB10* is involved in anthocyanin accumulation (Yu et al., 2019). Therefore, we conclude that *LvMYB5* plays an indispensable role in the coloration of lily petals.

In this study, *LvMYB5* was found to activate ANSp by binding to *cis*-acting elements in the –1,200 to –900 bp region. We further confirmed that *LvMYB5* is an activator of anthocyanin biosynthesis. Our results are in accordance with the results of studies on *Primulina swinglei* (Gesneriaceae) flowers showing that the TF PsMYB1 can activate the *PsANS* promoter to regulate anthocyanin biosynthesis (Feng et al., 2020). In addition, transient overexpression of the *MYB2* gene of *Dendrobium bigibbum* in *N. benthamiana* leaves also increased gene expression, which in turn promoted flower coloration (Lim et al., 2020). LrAN2-like, which binds to the *LrDFR* and *LrANS* promoters, can promote anthocyanin synthesis in *Lycium ruthenicum* (Li et al., 2020). Thus, MYB TFs can also simultaneously activate a variety of structural gene promoters to increase the accumulation of anthocyanins (Wang et al., 2019). In future research, we will isolate additional structural gene promoters for further gene regulation studies.

### Multiple MYB Activators and Repressors Collaboratively Regulate Anthocyanin Accumulation in Lily Petals

In this study, normal *N. benthamiana* leaves have no obvious accumulation of anthocyanins, and the expression of endogenous anthocyanin biosynthesis genes is also at a low level. Transient overexpression of *LvMYB5* up-regulated the expression of *NbCHS, NbDFR*, and *NbANS*, and promoted the synthesis of anthocyanins in *N. benthamiana* leaves. When *LvMYB5* and *LvMYB1* were co-expressed in *N. benthamiana* leaf epidermal cells, compared to the empty vector, expression of *NbCHS, NbDFR*, and *NbANS* was increased and the leaves contained a small amount of anthocyanin. It is possible that this was due to the combined action of an activator and an inhibitor, that *LvMYB5* promoted anthocyanin synthesis and *LvMYB1* inhibited anthocyanin synthesis to some extent. Compared to the 35S:*LvMYB5* construct, the expression of *NbCHS, NbDFR*, and *NbANS* in *N. benthamiana* leaves infiltrated with both the 35S:*LvMYB1* and 35S:*LvMYB5* constructs was significantly reduced, and there was very little anthocyanin in the leaves. Therefore, *LvMYB1* inhibits the anthocyanin biosynthesis pathway to a certain extent. The regulation of the anthocyanin biosynthesis pathway is complex, and it may involve multiple activators and inhibitors. This result is similar to a study of sweet potato leaves, where three MYB activators and five MYB repressors were found to be involved in the process of juvenile red fading in sweet potato leaves (Deng et al., 2020b).

After agroinfiltration, the lily petals became curling and slightly faded. In fact, the petals infected by agrobacteria carrying the empty vector displayed fading color which was lighter than normal petals (Supplementary Figure 3). Under the condition of the same growth environment, the reduced pigmentation caused by the agrobacterium inoculation may be due to the stress caused by the agroinfection. The same color fading was observed in *Nicotiana benthamiana* leaves, peach fruits, and pear fruits in agroinfiltration experiments (Yao et al., 2017; Zhao et al., 2020). Nevertheless, the color of the petals transiently expressing 35S: *LvMYB5* was significantly darker than the petals infiltrated with the empty vector. Our results are consistent with a positive role of *LvMYB5* in promoting color formation, whereas *LvMYB1* may inhibit the coloration of lily petals.

In addition, previous studies on lily have shown that LhMYB12, LhMYB12-lat, LrMYB15, and LhMYB18 can regulate anthocyanin pigmentation in the entire petal, the ovary, the flower bud, and the large anthocyanin spot, respectively, which promoted lily petal coloration (Xu et al., 2016; Yamagishi, 2016, 2018). However, functional verification tests need to be improved. In this study, we characterized LvMYB5, a member of the SG6 subgroup of MYB positive regulators that activate the anthocyanin biosynthesis pathway. The C-terminal sequence in LvMYB5 differs from those in other MYB proteins (Supplementary Figure 4), which may be due to species-specific differences (Yin et al., 2020). Recently, R3-MYB inhibitors have also been reported from lily (Sakai et al., 2019), and these inhibitors may be derived from n-terminal truncation of SG4 subgroup MYB proteins (LaFountain and Yuan, 2021). In this study, we found that LvMYB1, which belongs to the SG4 subgroup, inhibits anthocyanin synthesis. MYB TFs in this family have negative impacts on anthocyanin accumulation by competitively binding bHLH TFs in the MBW complex, or through the inhibitory function of the EAR motif or the TLLLFR inhibitory motif (Fornale et al., 2010).

In this study, the transient over-expression of *LvMYB1* in lily flower petals caused the petal color to fade. Thus, *LvMYB1* appears to play an inhibitory role in anthocyanin biosynthesis in lily flower petals. Also, a multiple sequence alignment that included LvMYB1 and LvMYB5 showed that K (lysine) was substituted for L (leucine) in the bHLH binding site of LvMYB1. The L at this position was conserved in all other MYB proteins included in the analysis, regardless of whether they were transcriptional activators or repressors. Therefore, the combination of LvMYB1 and bHLH *in vivo* is likely to be affected, and LvMYB1 would not affect anthocyanin synthesis through competitive binding with a bHLH TF.

LvMYB1 contains a typical inhibitory sequence, the ethylene response element binding factor (EAR) motif, which plays an inhibitory role in anthocyanin biosynthesis (Deng et al., 2020a). Previous studies have shown that lack of an EAR motif leads to reduction or loss of the inhibitory function in MdMYB16 (Xu et al., 2017). In addition, EAR motifs have been shown to convert transcriptional activators into repressors in chimeric protein fusion experiments (Matsui et al., 2004; Kasajima and Sasaki, 2016). Thus, the EAR motif is the main functional motif in MYB transcriptional inhibitors. Therefore, our preliminary results show that LvMYB1 can inhibit the synthesis of anthocyanins, although the inhibitory mechanism needs further exploration.

## Data Availability Statement

The raw data supporting the conclusions of this article will be made available by the authors, without undue reservation.

## Author Contributions

LJC conceptualized the project. XJY and YBZ carried out the laboratory work and data analysis and wrote the first draft of the manuscript. BHW, YDZ, and MI helped to review and edit the manuscript. YLF has been involved in critically revising the manuscript for important intellectual content. LJC and LZ had overall responsibility for this project, including project ideas, guidance on experimental design, data analysis, and manuscript writing and revision. LJC took part in the project administration and funding acquisition. All authors have read and approved the final manuscript.

## Conflict of Interest

The authors declare that the research was conducted in the absence of any commercial or financial relationships that could be construed as a potential conflict of interest.

## Publisher’s Note

All claims expressed in this article are solely those of the authors and do not necessarily represent those of their affiliated organizations, or those of the publisher, the editors and the reviewers. Any product that may be evaluated in this article, or claim that may be made by its manufacturer, is not guaranteed or endorsed by the publisher.

## Footnotes

1
http://bioinformatics.psb.ugent.be/webtools/plantcare/html/

2
https://www.dna.affrc.go.jp/PLACE/?action=newplace

## Supplementary Material

Supplementary Figure 1 The sequences of *LvMYB5*.

Supplementary Figure 2 Expression analysis of *LvMYB1*. **(A)** Transient overexpression of *LvMYB1* in *Nicotiana benthamiana* leaf cells. **(B)** Extraction of total anthocyanins from *Nicotiana benthamiana* leaves in the transient overexpression experiment. **(C)** Quantification of anthocyanin content in *Nicotiana benthamiana* leaves infiltrated with the empty vector or 35S:*LvMYB1.* **(D)** Expression levels of anthocyanin biosynthesis structural genes in *Nicotiana benthamiana* leaves.

Supplementary Figure 3 Transient overexpression experiments on lily petals. **(A)** Before agroinfiltration, lily bud (about 10 cm) and the outer petals, which were green and had just begun to color. **(B)** After agroinfiltration (1 week later), the lily petals infiltrated with the empty vector became curling and slightly faded. Red box: the region of the petals infected by agrobacteria; Black arrow: uninfected petal region.

Supplementary Figure 4 Multiple-sequence alignments of LvMYB5 with MYB related-anthocyanin biosynthesis TFs in other lily cultivars.

Supplementary Table 1 The sequences of oligonucleotide primers used in this study.
