# Supplementary figures and images for: Regulation of MYB Transcription Factors of Anthocyanin Synthesis in Lily Flowers
Source: Front Plant Sci. 2021 Dec 1;12:761668. doi: 10.3389/fpls.2021.761668 (PMC8672200; doi:10.3389/fpls.2021.761668)

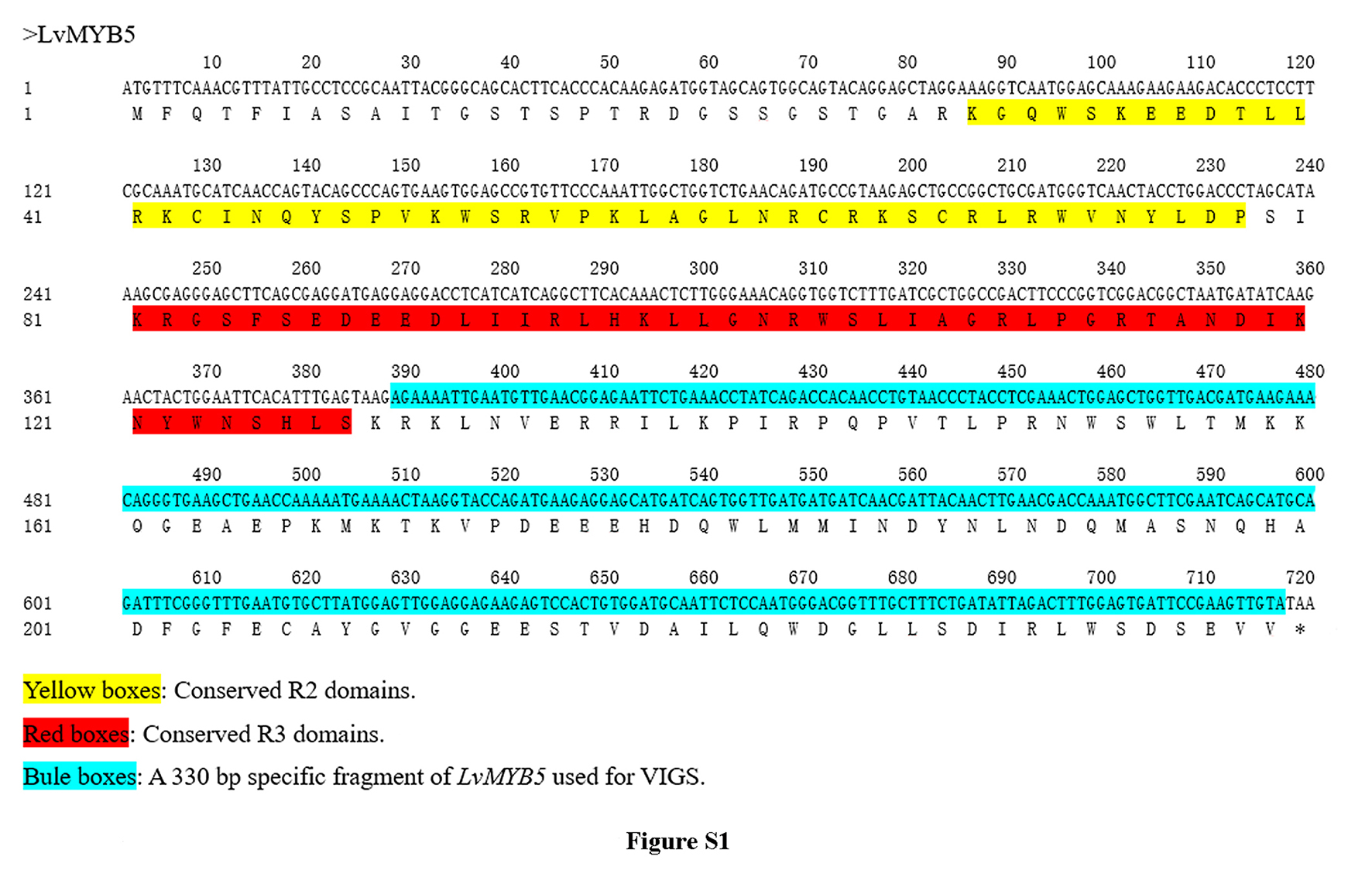

Supplement: Supplementary Figure 1 — The sequences of LvMYB5. [file Image_1.JPEG]

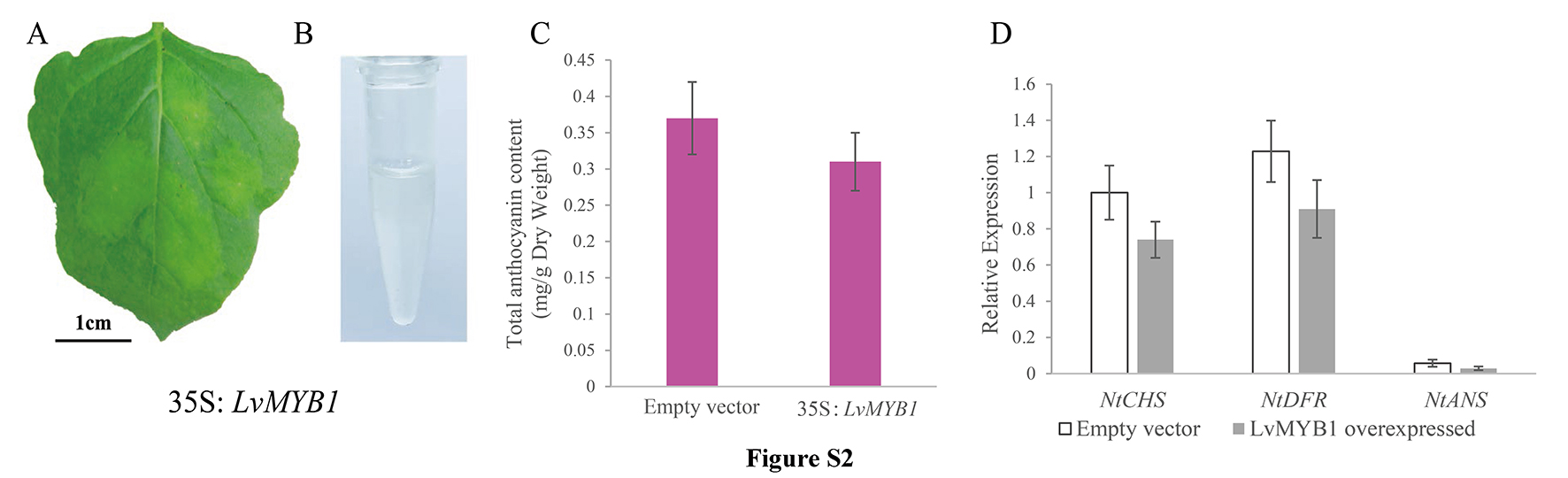

Supplement: Supplementary Figure 2 — Expression analysis of LvMYB1. (A) Transient overexpression of LvMYB1 in Nicotiana benthamiana leaf cells. (B) Extraction of total anthocyanins from Nicotiana benthamiana leaves in the transient overexpression experiment. (C) Quantification of anthocyanin content in Nicotiana benthamiana leaves infiltrated with the empty vector or 35S:LvMYB1. (D) Expression levels of anthocyanin biosynthesis structural genes in Nicotiana benthamiana leaves. [file Image_2.JPEG]

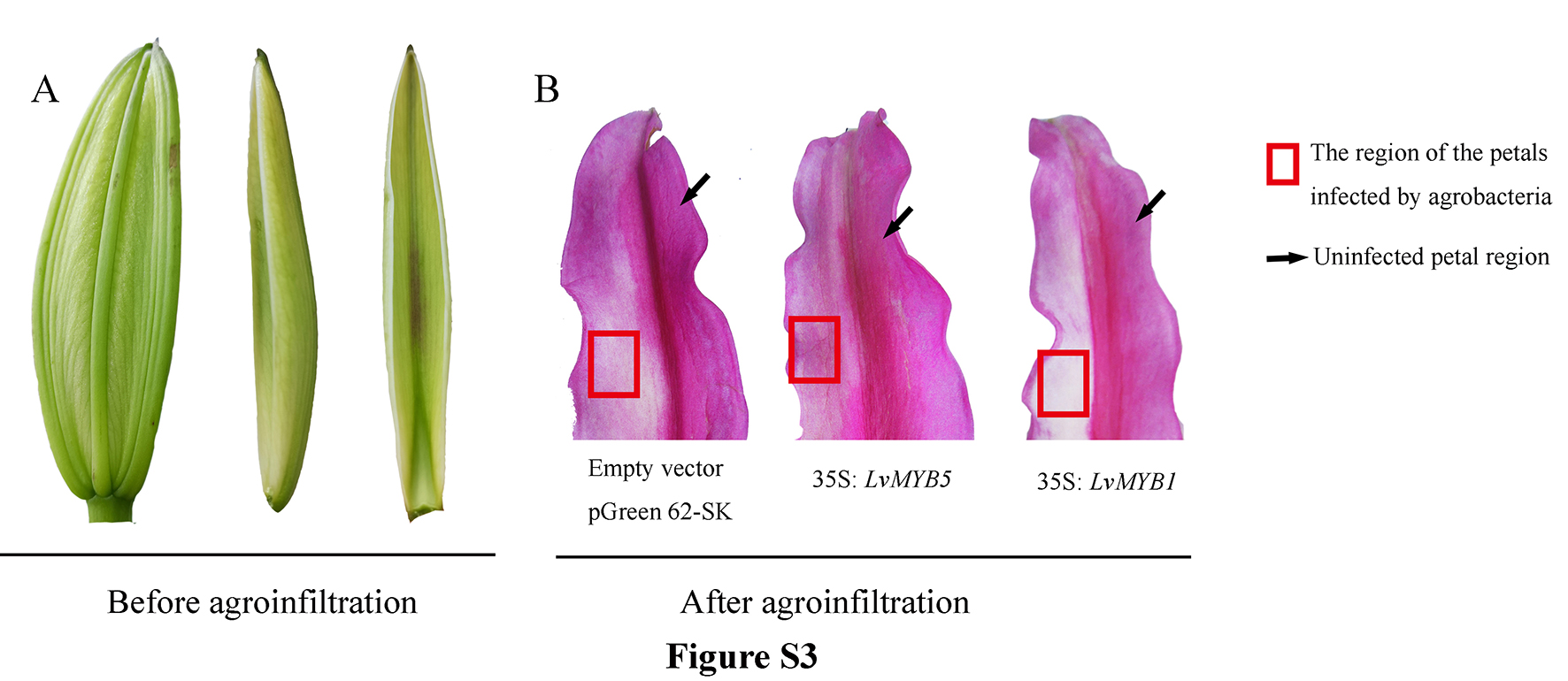

Supplement: Supplementary Figure 3 — Transient overexpression experiments on lily petals. (A) Before agroinfiltration, lily bud (about 10 cm) and the outer petals, which were green and had just begun to color. (B) After agroinfiltration (1 week later), the lily petals infiltrated with the empty vector became curling and slightly faded. Red box: the region of the petals infected by agrobacteria; Black arrow: uninfected petal region. [file Image_3.JPEG]

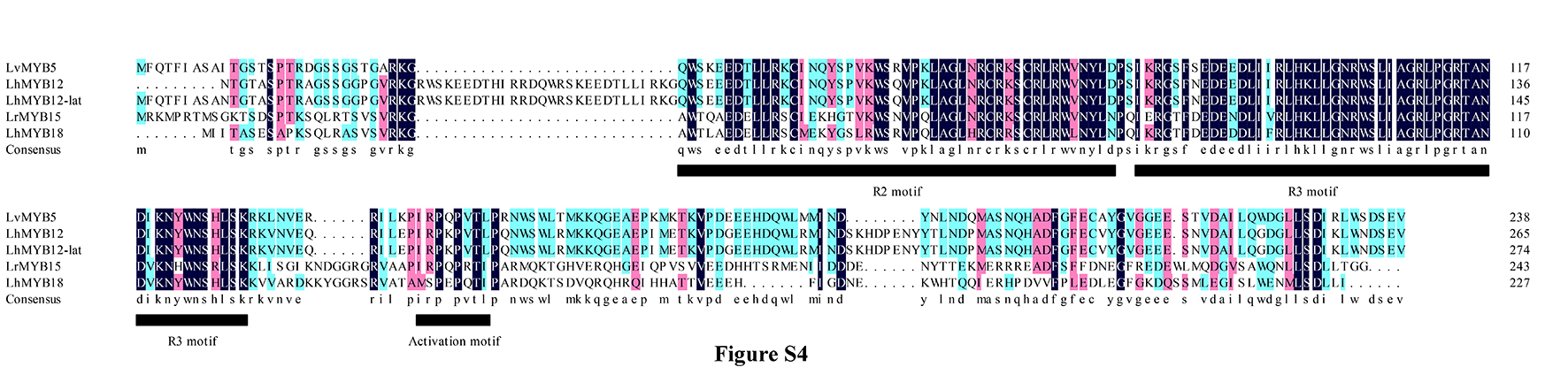

Supplement: Supplementary Figure 4 — Multiple-sequence alignments of LvMYB5 with MYB related-anthocyanin biosynthesis TFs in other lily cultivars. [file Image_4.JPEG]
