# Supplementary material for: Regulation of MYB Transcription Factors of Anthocyanin Synthesis in Lily Flowers
Source: Front Plant Sci. 2021 Dec 1;12:761668. doi: 10.3389/fpls.2021.761668 (PMC8672200; doi:10.3389/fpls.2021.761668)
Supplement: Supplementary Table 1 — The sequences of oligonucleotide primers used in this study. [file Table_1.DOCX]

**Supplementary Table S1** | The sequences of oligonucleotide primers used in this study.

| **Number** | **Premier** |
| --- | --- |
| 1 | *MYB1-*F: TACCAGCATAATATGGTAAAC |
| 2 | *MYB1-*R: CTCATGGGTAGAGCTCCTTG |
| 3 | *MYB5-*F: ACTACAATGTTTCAAACGT |
| 4 | *MYB5-*R: CCACCATATTATACAACTTCGG |
| 5 | *LvMYB5*F: CGGGATCCATGTTTCAAACGTTTATTGC（*BamHI*） |
| 6 | *LvMYB5*R: CCGGAATTCTTATACAACTTCGGAATCAC（*EcoRI*） |
| 7 | *LvMYB1*F: CGGGATCCATGGGTAGAGCTCCTTGTTG（*BamHI*） |
| 8 | *LvMYB1*R: CCGGAATTCTCATAGACTCACGCCCTCAT（*EcoRI*） |
| 9 | *LvMYB5*-VIGSF: GGAATTCAGAAAATTGAATGTTGAACG（*EcoRI*） |
| 10 | *LvMYB5*-VIGSR: CCGCTCGAGTACAACTTCGGAATCACTCC（*XhoI*） |
| 11 | *ANSp*-LucF: ACGCGTCGACGCCCGGGCTGGTATCACTTTATC（*SalI*） |
| 12 | *ANSp*-LucR: CCCAAGCTTCATGGAGCTTGGTTTGGTTGA*（HindIII）* |
| 13 | *ANSp*1-LucF: ACGCGTCGACCAGTATCGCTCATGGTGATC（*SalI*） |
| 14 | *ANSp*2-LucF: ACGCGTCGACCTCTCCCCTTTTTTTAGAGGAG（*SalI*） |
| 15 | *ANSp*3-LucF: ACGCGTCGACTGGACCGAAATCATGAGAGC（*SalI*） |
| 16 | *LvMYB5*-GFPF: GGGtaCCATGTTTCAAACGTTTATTGC（*KpnI*） |
| 17 | *LvMYB5*-GFPR: GCTCTagATACAACTTCGGAATCACtcc （*XbaI*） |
| 18 | *LvMYB1*-GFPF: GGGTACCATGGGTAGAGCTCCTTGTTG （*KpnI*） |
| 19 | *LvMYB1*-GFPR: GCTCTAGATAGACTCACGCCCTCATTG （*XbaI*） |
| 20 | *LvCHSF*: GGAGGGCAAGGCAACCAC |
| 21 | *LvCHSR*: GCAGCAGCAAACCATTAGGC |
| 22 | *LvDFRF*: GGGTCCTTTCATCACCTCAAC |
| 23 | *LvDFRR*: CCACTCGCTTCTGGATTCTCA |
| 24 | *LvANSF*: CCAAATGTCCGCAACCTGA |
| 25 | *LvANSR*: GCCAATGTGGACGAGAAGC |
